# A’uwẽ (Xavante) social constructions of well-being in Central Brazil

**DOI:** 10.1080/01459740.2021.1961247

**Published:** 2021-08-12

**Authors:** James R. Welch

**Affiliations:** Escola Nacional de Saúde Pública Sérgio Arouca, Fundação Oswaldo Cruz, Rua Leopoldo Bulhões 1480, Rio de Janeiro, RJ, Brazil

**Keywords:** health, social relations, living well, suffering, environment, Brazil

## Abstract

Well-being is a heterogeneous idea with inconsistent applicability to real-world circumstances. In this article, I explore A’uwẽ (Xavante) notions of social well-being from an ethnographic perspective. My data indicate many members of this Indigenous group understand wellness to involve not only health and harmony, but also certain modes of strife and inequality that are also viewed as desirable. A’uwẽ understandings of social wellness, including linkages to the environment, suggest that a broader and more locally contingent concept of social well-being than is evident in mainstream literature would benefit transcultural health efforts and policy involving Indigenous and other culturally distinct communities.

Well-being, a seemingly common-sense concept closely allied with health, defies attempts at definition, qualification, and quantification in transcultural perspective. Rooted in the apparently simple condition of being well, it is a heterogeneous idea that provokes intriguing questions about its applicability to plural real-world circumstances, the role of disciplinary boundaries in framing its meanings, and how to reconcile myriad quantitative indicators that purport to capture its essential dimensions. Such a seemingly simple term provokes widely diverse academic interpretations in part because it involves multiple and often divergent facets that reach well beyond mainstream quality of life measures into other arenas, including social life as locally understood.

The constellation of ideas coded with the term well-being come from a variety of disciplines including philosophy, psychology, economics, sociology, epidemiology, and public health. They include both “subjective” and “objective” definitions, which are distinguished by how they engage with individual perception versus external verifiability (Sumner 1996). They may also be distinguished by whether they are hedonic or eudaimonic, the former being closest to happiness and the later indicating life lived fully and satisfyingly (Deci and Ryan 2008). Multiple pursuits to define and measure well-being are well underway but have not neared their conclusion, in part because of disciplinary crosstalk but also because of its relevance to public policy, which requires ideas with identifiable links to resources and services within the public purview (Diener et al. 2009).

All academic fields contributing to the well-being discussion have their own epistemological assumptions about appropriate methods for producing knowledge, most of which are very different from anthropology’s. Among the unique contributions of ethnographic studies are robust and holistic approaches that prioritize the diversity of local understandings. Ethnographic approaches ask how different cultures engage their own concepts of wellness and what work others need to do to understand them. Thus, they tend to focus on aspects at the subjective, eudaimonic, and qualitative ends of the spectra. They also show greater interest in sociocultural than individualistic dimensions of well-being, while recognizing that these dimensions overlap and engage one another (6, Perry. 2007).

Psychologists have argued that well-being is social and cultural as much as it is individual (Prilleltensky and Prilleltensky 2007). Similarly, within the field of public health, good health is understood as “not merely the absence of disease, but a state of complete physical, mental, spiritual and social well-being” (WHO 1948). In this article, I use an ethnographic lens to explore A’uwẽ (Xavante) notions allied with the term well-being from a social perspective. As I explain in subsequent sections, my reading of A’uwẽ thinking led me away from emphasizing narrow numerical indices intended for comparative purposes in favor of a broad interpretation of well-being as continually socially constructed. I also do not endeavor to explore the full range of A’uwẽ ideas that bear upon emic notions of well-being. My viewpoint of A’uwẽ society was strongly colored by the emphasis my hosts and consultants placed on certain aspects of social life. I therefore focus on ascertaining how A’uwẽ people may view “well-being” with regard to interpersonal social arrangements rather than cosmological and ontological schemata that have been a major focus of anthropological thinking about Indigenous peoples in lowland South America in recent decades (Fischer 2018; Ramos 2012; Rival 2012). I discuss how A’uwẽ notions led me to a particular anthropological formulation of social well-being that I find productive for understanding their case and promising for contributing to transdisciplinary and transcultural discussions about Indigenous and other culturally distinct peoples’ health.

The study group included four principal A’uwẽ communities located in the Pimentel Barbosa Indigenous Land, State of Mato Grosso, Central Brazil, a region of cerrados or tropical savannas known for their exceptional but threatened biodiversity (Strassburg et al. 2017). Between 1968 and 1972, the Pimentel Barbosa community was established after its approximately 200 founding residents were coerced by government agents to settle within the current borders of the Indigenous land (Welch et al. 2013). I began fieldwork in 2004 and have subsequently visited on average once or twice per year. In 2006, Etênhiritipá separated from Pimentel Barbosa due to internal political conflicts, relocating just a half kilometer away. Two other villages, Novo Paraíso and Santa Vitória, subsequently split from Pimentel Barbosa. Today, Pimentel Barbosa and Etênhiritipá have approximately 400 and 500 residents, respectively, while Novo Paraíso and Santa Vitória have a little over 100 residents each. There are now another 12 communities in the Indigenous land, which were not participants in my primary ethnographic studies.

## Antônio

I begin this article with a profile of my late adoptive A’uwẽ grandfather Antônio (Ru’wẽ’warazu’ata) and how his unique life and personality illustrate some of the dilemmas and solutions involved in assessing well-being from anthropological, public health, and other academic frameworks. I met Antônio as soon as I first arrived at Pimentel Barbosa in 2004 and came to know him by working with him in his gardens, accompanying him on hunts, and whiling the time away in conversations about any number of topics.

Antônio was an agriculturist, a hunter, a fisher, and often a collector of fruit, roles closely linked to his identity as a husband, father, and grandfather, who proudly cared for his family along with his wives and his mother-in-law. Acquiring game meat and fish were mostly his domain as the senior male member of this small group of household caretakers. He was a determined and knowledgeable hunter, one of the last in the study communities to use a bow and arrows.

Antônio had not adopted the conservationist language acquired by several younger A’uwẽ men while living in metropolitan cities and engaging with national and international environmental conservation agencies. Antônio worked on nearby ranches in the 1970s but had otherwise remained a resident of local A’uwẽ communities. His concern for the local environment was tangible and passionate, guided by his interest in obtaining resources from it over the long term. His understanding of how to preserve the landscape for the future was based on his life experience of extracting resources from it before, during, and after largescale encroachment and circumscription by cattle and monoculture agricultural ranches. He spoke to me about the landscape in very pragmatic terms, which communicated a strong sense of place, but without resorting to characterizations of it in animistic terms (Barletti 2016).

Among the first to invite me to accompany him for a day clearing weeds in his garden, he also was one of the first hunters to give me the opportunity to shadow him on group hunts, which were a special interest of mine (Welch 2014; 2015). On these hunts and during our conversations, Antônio demonstrated to me his deep knowledge of and concern for the landscape, and its incredible diversity of plants and animals. He considered a healthy and conserved landscape essential for extracting resources from their reduced territory in the present and future, and thereby maintaining what he characterized as traditional values involving provisioning one’s family with wild and cultivated foods, sharing these foods with one’s extended family and neighbors, and giving gifts of healthful foods at designated moments during some of life’s most important ceremonial events.

**Figure 1:**
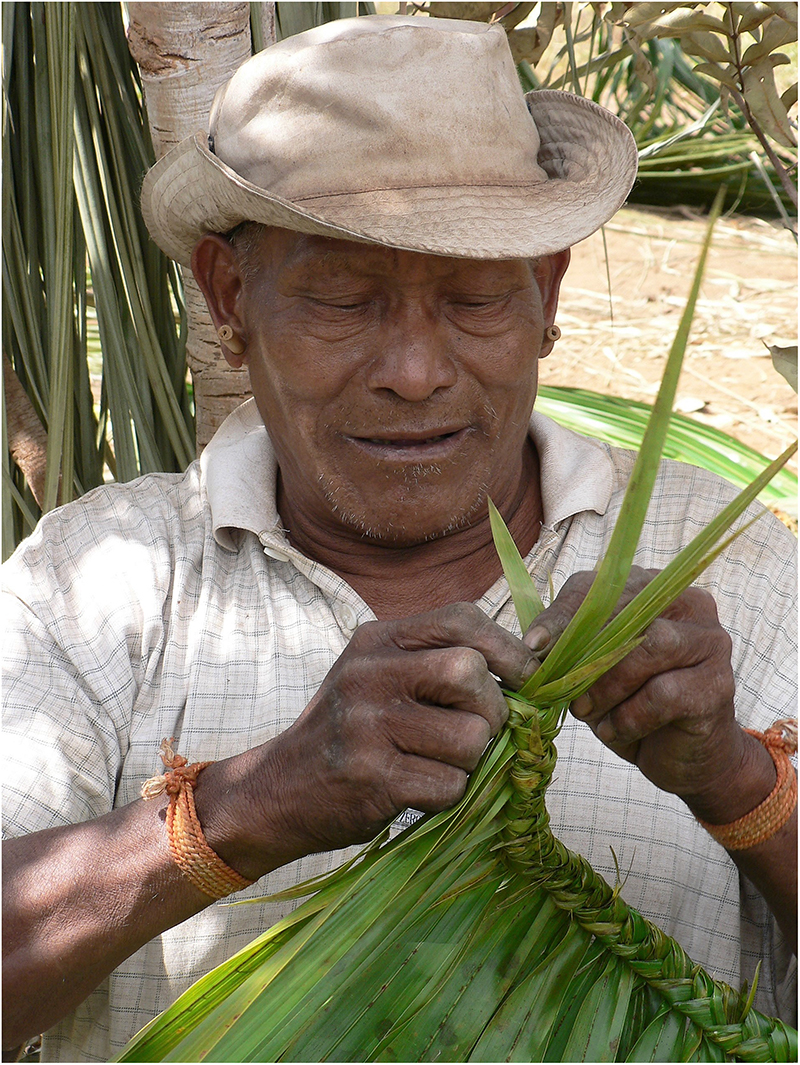
Antônio weaving a ceremonial mantle in his role as elder owner and leader of the Tebe (Fish) ceremonies during rites of initiation of pre-initiate boys into novitiate adulthood. Photography by James R. Welch, 2006.

Antônio and his wives, with the assistance of his mother-in-law, cared for their fourteen rowdy children with all the love, pride, and hard work one could imagine. In community ceremonial life, he performed an essential role as the elder owner and leader of the Tebe (Fish) ceremonies (*tepé’tede’Wa*), which are an important component of the ceremonial complex that makes up the rites of initiation of girls and boys into novitiate adulthood (Figure 1). He also served as a post-officiant (*wai’a’rada*) during spiritual rituals, giving advice and critique to the younger individuals who were now responsible for carrying them out. In secret, he was the senior owner of a series of heritable prerogatives that he employed for the benefit of his family and the community at large. I considered Antônio to exemplify social well-being in this A’uwẽ community.

Antônio (and other elders who paid me special attention over the years, many of whom are now deceased) looked after me in his own individual caring ways. His concern for me as a guest in the community was not exceptional for him (it was for me) and illustrated A’uwẽ capacities for creating special affective bonds with non-A’uwẽ who show an interest in their community and well-being. Among the ways they did this was including outsiders in adoptive families, and thereby exogamous moieties, as well as age sets, and thereby age set moieties, in order to orient them socially to the whole of society and give A’uwẽ residents a basis for approaching them properly in socially proximate or distant ways, or with culturally appropriate affinity or antagonism.

Antônio’s and other’s interest in outsiders spoke to me about their concern with their own families’, communities’, and ethnic group’s welfare in a rapidly changing world of territorial circumscription, market insertion, transcultural engagement, biomedical healthcare services, and social media. By engaging outsiders like myself, these elders sought to enhance their community’s sovereignty amid upheaval in diverse realms. This sovereignty derived from building knowledge and networks beyond the community that could reinforce internal sources of resilience. As has been mentioned elsewhere, in many ways we are “their” anthropologists rather than the other way around (Welch and Coimbra Jr. 2014).

Antônio died after several years of major paralysis resulting from a stroke. His last years of life were filled with deep emotion as he tried but could no longer communicate well verbally with his loved ones and friends, including myself. Although his family cared for him attentively and close friends visited regularly, his wheelchair broke repeatedly, his clothes fell into a state of disrepair, and his evident desire to be close to other people went insufficiently attended as community life continued without his usual active participation. When his wheelchair worked, he spent much of his time in front of his house, where he could see the entire community plaza and all the activities that occurred there. When his wheelchair was broken, which was often, he spent most of his days in his kitchen annex behind the house, where he could enjoy the company of members of his extended family household and other visitors as they came and went.

His suffering could be easily interrupted with the pleasure of a friendly visit, a warm hand on his knee, a hug, or a cathartic cry with someone close. Antônio was ill, but he was also well in so many ways. His health was poor, but his depth of feeling and connection with others was rich and full until he died. Whereas some scientific or popular formulations of well-being might characterize him as disadvantaged, I understood his degree of wellness to be very high despite his stroke sequelae. This was because his A’uwẽ relatives and peers saw him as the same loving person who abundantly contributed to his family and community with all the responsibility and care that are expected of a husband, father, grandfather, elder, and ceremonial prerogative owner. In his final years, his person was not defined exclusively by his paralysis, but also by his life-long successes, contributions, and affections.

Antônio’s example illustrates why I am led by my ethnographic data to focus on well-being as construed very openly, especially including many social aspects of wellness besides physical and mental health (Steckel 2016). Health is a very important dimension of well-being. Yet, as Mathews and Izquierdo (2009:4) note, “health and well-being may contract one another.” There is no doubt that Antônio suffered in his final years, but he suffered within the fullness of a life lived robustly in connection with his family, community, non-A’uwẽ friends, local landscape, and ceremonial responsibilities.

I am not the first to call for a broad ethnographic understanding of well-being. For example, Barletti (2016) made a similar argument based on ethnographic research with the Ashaninka people in Peru, with particular focus on perceptions related to cosmological ontologies. Based on ethnographic work with the Matsigenka in Peru, Izquierdo (2005) sought to expand the notion of well-being to include cultural formulations of goodness and harmony including spiritual realms, which may suffer even as biomedical measures improve. Panelli and Tipa (2009) discussed Maori concepts of well-being involving sociocultural and environment dimensions integrated with spiritual elements. Because spiritual topics are closely held secrets among the A’uwẽ that cannot be shared with the public, and not having found ontological interpretations particularly ethnographically pertinent among the A’uwẽ, my contribution is to show how productive formulations of well-being can involve local social values of the kinds illustrated by Antônio’s story, such as indulging affection and suffering together.

## Traditionalism and social well-being

Within A’uwẽ communities, overwhelming support exists for the maintenance of certain challenging customary rites of passage, especially those related to two age group systems closely intertwined with social and ethnic identity, as described below. Thus, most A’uwẽ support cultural traditionalism in at least some contexts, even if younger people’s attentions are not particularly directed towards the preservation of all other customs. I use the terms traditionalism and traditionalist to capture emergent emic notions of contemporary advocacy of culture and customs considered “traditional” (*wahöimanazé*) by A’uwẽ people. I do not intend for these terms to imply religious conservatism specifically or to suggest any negative connotations such as antiprogressive thinking. Nor do I mean to suggest that culture is static, although some A’uwẽ perspectives of tradition involve ideas about an idealized past.

Age group systems, a major focus of popular A’uwẽ traditionalism, are formed by the highly formalized intersection of age sets (cohorts) of individuals that pass through age grades (phases of life) by means of collective public rituals. In other words, age grades are formalized through their occupation by sequential age sets, which occurs ceremoniously for members of an age set at the same time (Bernardi 1985). In the A’uwẽ case, adjacent age sets belong to opposite age-set (agamous) moieties while alternate age sets belong to the same moiety. An ethos of camaraderie and permissiveness is shared among members of the same moiety, while between members of opposite moieties a sense of hierarchical antagonism and competition predominates.

Interestingly, this same format applies to two distinct age group systems, one which applies both to men and women and is considered by the A’uwẽ to have a secular focus, and one which includes only male members and is considered fundamentally spiritual in nature. Although the first of these two systems involves both women and men, through the male-only pre-initiate house experience (up to approximately five-year residence in symbolic isolation from the community) it engenders special relationships of camaraderie between male age set peers and their mentors (members of the next oldest age set in the same age-set moiety, on average about 10 years older than their protégés). The second is a secret men’s cult and cannot be discussed in detail with women or outsiders. Nor may its secret activities and contents be published in this article. These social configurations are important examples of culturally appropriate expressions of differentiation and ranking contributing to a thoroughly plural social tapestry of contrasts between people that is fundamental to traditionalist perspectives of how a good life is lived (Welch 2009; 2010).

**Figure 2:**
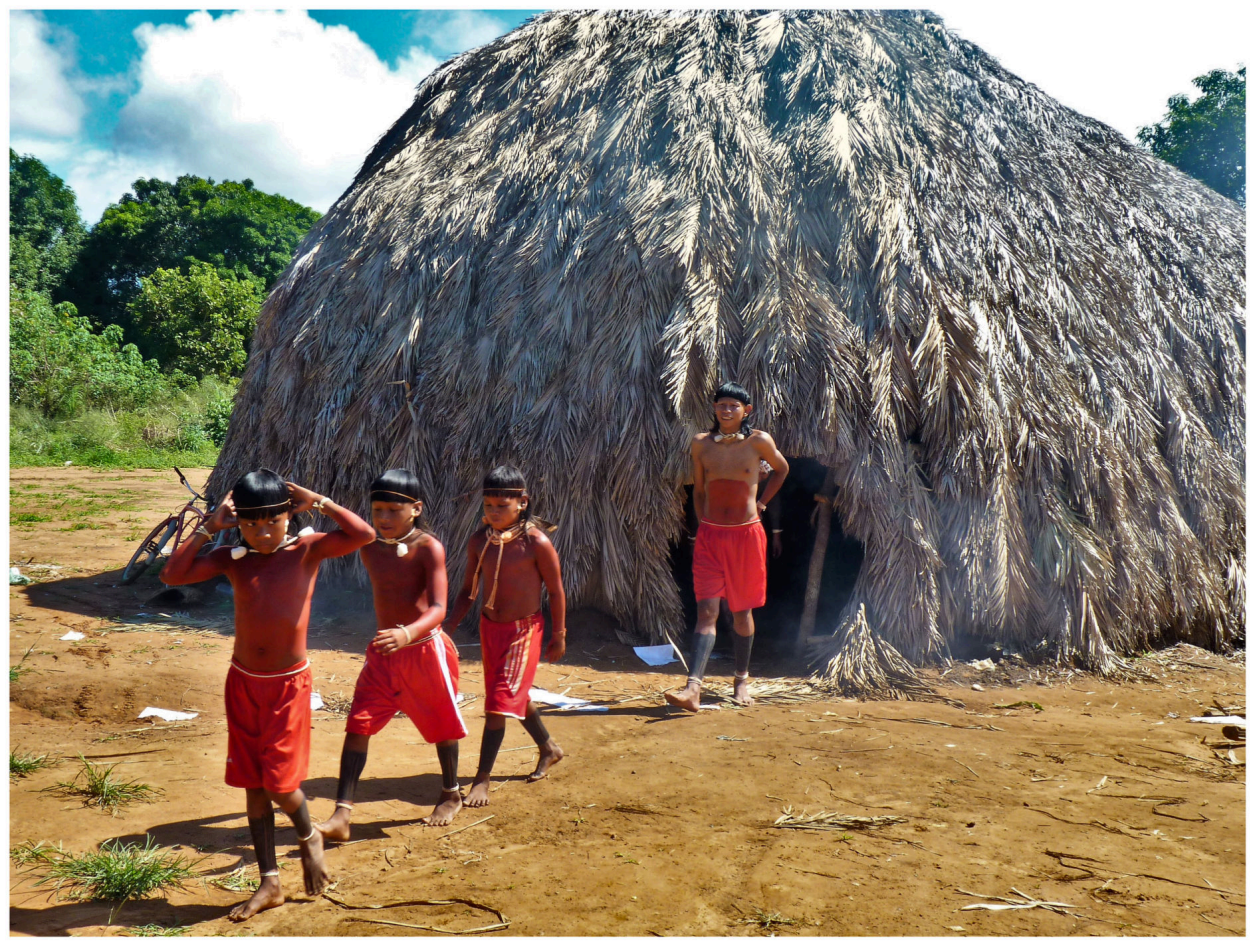
The pre-initiate boys’ house (*hö*) in Pimentel Barbosa community. Photography by James R. Welch, 2011.

My pre-initiate male interviewees affirmed enthusiastically that their experience in the pre-initiate house (Figure 2) was unchanged from the time their ancestors and grandparents lived in this house during their own youth. They considered this continuity a source of great pride. Elder men tended to agree with these youths, asserting that the pre-initiate experience including residence in the pre-initiate house under the indulgent guidance of their mentors was a characteristically A’uwẽ format for constructing men from boys that remained unchanged through history. However, at other times and in other contexts, these same elders also expressed that the pre-initiate experience was diminished today and was likely of little value compared to their own era. With regard to the spiritual age group system, which is paced on a 15-year cycle of initiation rites (taking up to 45 years for a male to complete the entire sequence of spiritual age grades), elder men expressed similar ambivalence to me with regard to its proper continuation and contemporary dilution.

I interpreted these seemingly contradictory narratives as deriving from the simultaneous fallibility of the individuals engaged in these age group systems (especially protégés and their mentors) and the irreproachability of the social arrangements (mentorship relations derived from the intersection of formal age grades and age sets) perpetuated through the generations. Thus, the social configurations involved in mentorship remain intrinsically good and beautiful, while the individuals involved in them may fall short of expectations. These irreproachable social arrangements are an excellent example of traditionalist values that are similarly shared by younger and older people. As I mentioned above, boys take great pride in asserting that their experiences in the pre-initiate house were unchanged from the past. Notably, all boys opt to live with their secular age set peers away from home for up to about five years rather than stay at home, even though A’uwẽ notions of child autonomy (Idioriê 2019; Nunes 2011; Welch 2015) give them the right to choose of their own accord. Also, female members of mentoring age sets are recently expressing new forms of agency by asserting their desire to participate in some formerly male-only secular age set ceremonies. With females now participating in some such ceremonies, the enthusiasm to participate among youths of both genders is tremendous.

I interpret the near universal enthusiasm to participate in age group system activities, especially diverse public ceremonies and rituals and excursions into the forest by mentors and their protégés, as indication that these social arrangements are an expressive focus of A’uwẽ traditionalism, anchored in the interests of the entire population, including women and men of diverse ages. Furthermore, they are widely understood to represent the only proper and good way of creating men from boys, contributing substantively to notions of ethnic belonging. Thus, it is my ethnographic understanding that these age group systems are examples of traditionalist social configurations amply believed to promote well-being among the population, both for those who participate publicly and those who help prepare behind the scenes and watch.

**Figure 3:**
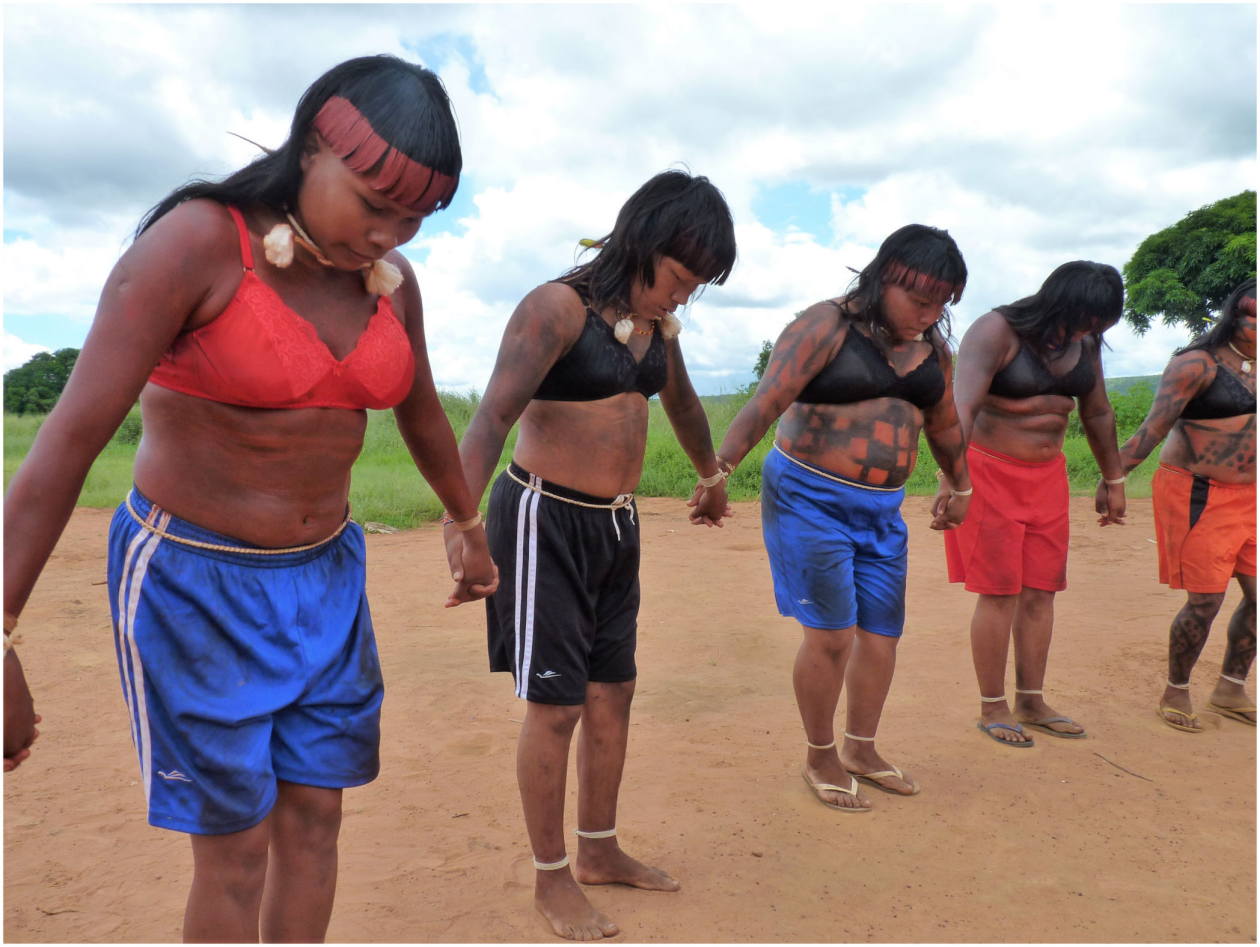
Women participating in age set singing performance (*danho’re*) in Pimentel Barbosa community. Photography by James R. Welch, 2011.

By joining a coed secular age set and living in the all-male pre-initiate house for years before advancing to novitiate adulthood, a boy is assuming his responsibility to do the internal and external work required to become a respectful and responsible adult who provides for his family and participates appropriately in the community. By identifying with her secular age set, assisting pre-initiates thatch their shelter, and participating in ceremonies recently opened to females (Figure 3), a woman is supporting her age-set protégés and peers and contributing to the beauty of age-set relations. By suffering with their protégés during public performances for many hours in the hot cerrado sun, coed mentors reinforce the vigor of their protégés and contribute to their successful assumption of mentorship roles a decade down the line. By enduring and reciprocating the rivalry of their immediate elder age set, which belongs to the opposite age set moiety, age set peers participate in a culturally sanctioned form of antagonistic oversight that serves to promote their appreciation for respect and duty. In all these examples, social well-being is expressed through age group systems, symbols of traditionalism amidst dramatic transformations in the circumstances of life for the A’uwẽ.

Age group systems are not the only example of social well-being expressed through traditionalism. Other examples include weddings (and associated wedding hunts), secret female knowledge regarding wild root vegetables, pregnancy and birthing practices, children’s play activities, men’s councils, and many more. What is unique about age group systems is the pervasive manner in which they condition the human experience from childhood to old age, thereby providing social bearings for all but the youngest living individuals, and women in the case of the spiritual age group system. Age group systems are ubiquitous aspects of daily social interaction through which meaning is constructed between individuals and groups of individuals (such as age sets and moieties).

## Well-being in anthropological perspective

According to Haworth and Hart (2007:1), well-being:

… has been viewed variously as happiness, satisfaction, enjoyment, contentment; and engagement and fulfilment, or a combination of these and other hedonic and eudaimonic factors. Well-being is also viewed as a process, something we do together, and as sense-making, rather than just a state of being. It is acknowledged that in life as a whole there will be periods of ill-being, and that these may add richness to life. It has also been recognized that well-being and the environment are intimately interconnected. Certainly, well-being is seen to be complex and multifaceted, and may take different forms.

Based on my ethnographic experience with the A’uwẽ, I identify with this characterization of well-being because it focusses on process and “sense-making,” as well as interconnectedness with such factors as the environment. Antônio’s sadness at the end of his life did not negate the fullness of his lifelong experience or cancel out his degree of contentment for having raised many children, hunted and gardened to provide them with food, and passed along secret knowledge to them. It did not undo the rich pleasure he expressed when reconnecting with those who had shared his life of generosity and goodwill. It did not betray his community’s respect for his years of ceremonial leadership and caring for his ceremonial protégés. In these ways, Antônio exemplified several A’uwẽ ways of “well-living” and “well-dying” (McGillivray 2007:29).

Based on these ethnographic observations, I seek to move beyond individualistic hedonic concepts of happiness, health, and wellness towards an understanding of A’uwẽ well-being as involving social notions of quality and richness of community life. I focus on aspects of social life, such as the age set systems, which my ethnographic research suggests many A’uwẽ understand to promote “the quality of their relationships with each other and the world, which, ideally, contribute to a deep and ensuring sense of intrinsic worth and existential certainty” (Eckersley 2011:633).

Wellness, well-being, living well, and good living are but a few of the popular academic terms for the intersection of factors that contribute to quality of life. Too often these concepts are reduced to oversimplified indicators of vital statistics, food security, socioeconomic status, developmental indices, health conditions, access to public services, happiness, psychological satisfaction, or social capital (although some studies seek considerable robustness in their use of such measures, e.g., Azzopardi et al. 2018). Each of these measures may reflect an important dimension of well-being (Godoy et al. 2005) but Antônio’s example shows us that quality of life is not easily reducible to any single factor or collection of numerical variables and should be understood as a complex process, incorporating the point of view (worldview) of the people of interest. Established quality of life indicators have been shown to be influenced by emic factors, which standardized cross-cultural instruments do not contemplate (Jenaro et al. 2005; Schalock et al. 2005). Recognizing the autonomy of Indigenous and other culturally distinct peoples to perceive well-being and its cultural determinants on their own terms and incorporating this diversity into research protocols and public policy are important steps in decolonizing healthcare. Other limitations and biases of mainstream well-being scholarship have been addressed in reviews from public health and anthropological points of view (Carlisle and Hanlon 2008; Thin 2009).

An important endeavor to investigate emic notions of public wealth or affluence as expressions of well-being was undertaken in the book *Images of Public Wealth: or the anatomy of well-being in Indigenous Amazonia*, edited by Fernando Santos-Granero (2015). Although my emphasis here is not on public wealth, my findings coincide with those of some of the authors of this important book. Among the A’uwẽ, well-being has to do with such notions as “good” (*wedi*), “beautiful” (*ĩwe*), “beautiful to see” (*ĩ’madö’öze*), and “respect” or “modesty” (*danhisé*). These terms are not unlike those proposed for the Gê group Mebêngôkre-Xikrin, for whom goodness and respect configure into notions of well-being (Gordon 2016). For the A’uwẽ, these terms suggest that among other dimensions, respect for traditional and therefore “good” and “beautiful” social relations are key to well-being.

Unfortunately, I did not directly ask Antônio how he understood quality of life or how he characterized his own well-being. He did make comments to me over the years that suggest ways he might have answered that question. For example, between bursts of tears during an enduring hug while sitting at the side of his deceased daughter’s body at her funerary mourning, he affirmed to me that I had earned my place in A’uwẽ life because I had suffered with the community. I had the sense he referred to the general suffering involved in living in his community with few of the comforts of city life, such as the hardship of pursuing game together without drinking water under the scorching sun in the sandpaper climate of the late dry season. I also understood him to be referring to the deeper social sufferings of community life, which in my experience included solitude and lack of privacy, along with the burdens of caring for my secular age group’s protégés, enduring numerous demanding rites of passage and other ceremonies, as well as occasional interpersonal strife. Considering the circumstances of our hug, I also believed him to be referring to the suffering of loss of life of loved ones, as this was not the first funeral I had attended during my fieldwork.

Sharing these sufferings brought us together through common experiences, and in that moment specifically through mourning his daughter’s death while embracing. I also understood him to be characterizing our friendship or adoptive kinship relationship as grandfather and grandson as based in something similar to Putnam’s “thick trust,” which is a basis for the strong bonds between close people that most contribute to social well-being (Putnam 2000:466). Examples such as this suggest the importance of robust A’uwẽ ideas like “suffering together” for peoples’ perceptions of good living. This viewpoint forefronts what is considered “‘good’ in the sense that it is meaningful and judged well by other people in accordance with social principles” (Thin 2009:31). I agree with Thin’s assertion that people tend to make meaningful distinctions between “feeling well” and “living a good life,” the latter being of greater interest considering my ethnographic findings.

I also agree with Prilleltensky and Prilleltensky (2007:57) that “contrary to prevalent notions that well-being is a personal issue, … it is also relational, organizational and communal.” While individuals may experience or share aspects of well-being, it is as communities and societies that they collectively construct shared notions of well-being. This is not to say that people agree on everything, but rather that they have the potential to share meaning through common experiences and viewpoints. My A’uwẽ friends are a heterogeneous group of people who also agree on many things, among them some aspects of what contributes to good living and satisfaction in life. Many of them explicitly recognize the distinction between individual and community values and understand that their representations of social wellness are influenced by different factors when speaking as a community representative as opposed to an individual resident. These stances may differ slightly or substantially, but do not imply contradiction.

A’uwẽ social arrangements, such as age group organizations and agamous and exogamous moieties, are emblematic dimensions of how A’uwẽ understand their own society and social values. They are more than anthropological constructions, being explicit dimensions of how A’uwẽ understand their own social identities, and essential to how they conceive of their unique ethnic identity. Furthermore, they are keystone components of traditionalism in contemporary A’uwẽ society, dovetailing with A’uwẽ notions of social wellness and good living.

Recent research into well-being in Amazonian societies has emphasized diverse dimensions, some of which align better than others with my approach to A’uwẽ well-being. Santos-Granero (2015:28) observes, “Well-being attains its maximum expression in the sentiments of happiness, beauty, and rejoicing aroused by collective action.” Conklin asserts for the Wari’ that collective well-being is related to the community’s abundance of food, productivity, vitality, and its ability to mobilize group activities and celebrations, as well as an ethos of caring for others (Conklin 2015). Among the Kïsêdjê (Suyá), well-being is about collective euphoria or rejoicing (Seeger 2015). Through well-organized large ceremonies, Kïsêdjê people achieve the satisfaction of euphoria, a sense of public wealth, associated with a notion of living morally well or beautifully together.

While these characterizations are similar in some respects to A’uwẽ notions of well-being, they lack a central aspect of my ethnographic argument, which is that some A’uwẽ constructions of social well-being involve certain modes of strife, opposition, and hierarchy, which are also viewed as good and proper. This point was well captured by Gordon, who made a similar argument for the Mebêngôkre-Xikrin, another Gê-speaking group, among whom differentiated social forms of resource and ceremonial goods ownership contribute to ethical and aesthetic concepts about what is “a good, beautiful and correct way life” (Gordon 2016:210). This author also suggests that among the Mebêngôkre-Xikrin, notions of well-being entail the maintenance of pervasive social differentiation, which is linked to ceremonial and other kinds of property. The A’uwẽ case is similar, where the expectation for “good” social relations involves age-related ranking, distinct prerogative ownerships by kin groups, and differentiated social roles in diverse aspects of ceremonial and every day social life. Gordon also expresses reservations about the universal applicability of the equivalence of conviviality and social well-being in Amazonia, which coincides well with my approach, whereby I highlight that A’uwẽ consider certain culturally appropriate and traditional forms of differentiation and ranking to be essential to living well.

The association between cultural traditionalism and well-being has been observed among Indigenous peoples elsewhere. For example, a study showed that among Aboriginal Australians, “attachment to traditional culture is found to be associated with enhanced outcomes across a range of socio-economic indicators” (Dockery 2010:315). For the Matsigenka in Peru, “questions about general health, well-being, and happiness usually evoked stories about an idealized golden past” (Izquierdo 2005:779). Among the A’uwẽ, social well-being depends on well-organized communities, which in turn require that individuals and families prioritize social behaviors and arrangements considered to be quintessentially traditional. These arrangements do not negate individualism in A’uwẽ society. Nor do they deny the reality of internal community conflict and occasional divisions. Rather, they place a burden on individuals to make thoughtful decisions considering traditional values for the sake of one’s own and one’s community’s well-being.

## Well-being without yardsticks

What form should an ethnographic study of well-being take to avoid the pitfalls of ethnocentric yardsticks and comparisons (Thin 2009)? There is substantial disagreement about the answer to this question. Some anthropologists argue that all comparison is futile while others assert that ethnography enhances the accuracy of cross-cultural comparisons (Colby 2009). Between these extremes are diverse approaches to discussing well-being in specific cultural contexts without overreaching the bounds of cultural relativism or universalism.

The approach I take here is to discuss well-being within a society on its own terms, as I understand it, without emphasis on quantifiable measures for external comparison. Another way of characterizing my approach is as an exploration of subaltern epistemologies of well-being in an A’uwẽ community. Thus, I avoid overemphasis on absolute standards of quality of life, such as psychological, socioeconomic, or demographic measures. My attention is directed towards social (Derné 2009) rather than physiopsychological dimensions of well-being, as I came to understand them through participant observation research at different times over the last decade and a half.

Well-being is more than an individual or internal state of being. One’s environment may also contribute to one’s wellness, happiness, and quality of life. This environment may be one’s social and physical surroundings, including everything from physical housing and sanitation conditions to home or community life and one’s greater urban or rural setting, including access to “natural” and cultural resources present in a local landscape (Adelson 2000). From an anthropological point of view, the kinds and dimensions of environments that may contribute to well-being differ from culture to culture and person to person. Whereas some studies emphasize social environments or ecologies, especially for their impacts on child well-being (Earls and Carlson 2001), other studies emphasize how dimensions of the “natural” landscape and ecology can affect diverse dimensions of quality of social life (Laird, Wardell-Johnson, and Ragusaf 2014). Barletti provides a particularly insightful discussion of links between well-being and “place as a position from which to produce knowledge of the world and experience it” (2016:44), which resonates well with A’uwẽ notions of ties between the local cerrado landscape and wellness.

The cerrado tropical savannah ecological landscape is not the only kind of environment one might address when discussing A’uwẽ well-being, but it is an often-overlooked dimension that was borne out in my research as central to well-being for many individuals and for communities more generally. Not only is the environment, understood as an anthropogenic landscape, a topic of frequent discussion with regard to self-provisioning and sustainable resource use, but it is considered a key link in communities’ abilities to meet social needs for gifts of collected, hunted, or cultivated foods on certain ceremonial occasions, which are some of life’s most important events. Game meat is given by grooms to brides’ households to formalize their weddings. Collected wild root vegetables and maize loaves are given by spiritual initiates to their spiritual singers, mentors who guide them in spiritual matters, during spiritual ceremonies. The link between environment, subsistence, and well-being among the A’uwẽ recalls the North American Cree, among whom well-being is less associated with health and disease and more closely aligned with the presence of game animals, food sovereignty, and maintenance of traditional values, including religious practices and native language use (Adelson 2000). In another example, Wolsko et al. (2006:353) report that “many participants expressed that this subsistence lifestyle is at the core of wellness for Yup’ik people [of southwestern Alaska], frequently referring to it as ‘the lifestyle’ or ‘the way of life’.”

Previous literature on the connection between well-being and the environment has focused on several themes that I do not prioritize specifically for the A’uwẽ for being outside the scope of this article or discordant with A’uwẽ ways of engaging the environment. Some studies emphasize the role of ecological services, landscape resources, and biodiversity on the economic quality of life or health of individuals (Dasgupta 2001). Others point to a correspondence between climate change or degradation of local ecosystems and poor living and sanitation conditions, leading to poor human health (Green and Minchin 2014). Another line of research addresses connection to “nature” as a positive factor for the well-being of urban residents (Bieling et al. 2014). Still another line of reasoning involves an association between traditional ethnobiological and ethnomedicinal knowledge and well-being, which has been documented among diverse Indigenous, traditional, and local groups (Johnson 2017).

I find several other approaches to the well-being/environment nexus more useful for the A’uwẽ case. For example, Richmond et al. (2005) identified reduced access to traditional territorial resources as one of three main factors impacting well-being among ‘Namgis First Nation. Studies of Aboriginal Peoples in Australia and Torres Strait Islanders have focused on the complex relationships between people and “Country,” a notion that encompasses constructive and destructive aspects, including physical, emotional, social, and spiritual dimensions of landscape and health (Butler et al. 2019). Panelli and Tipa advocate for drawing on Indigenous perspectives to expand upon the notion of foodscapes to better incorporate Indigenous perspectives of food as a nexus of diverse dimensions (“spiritual, physical, social, material, cultural, economic and political relationships”) that contribute to “being alive well” (2009:458). Other scholars advocate for similarly integrative approaches (e.g., Sangha et al. 2015), including Burnette et al. (2018:369) who address “how subsistence living may contribute to well-being and resilience by promoting physical exercise, a healthy diet, and psychological health.” In the A’uwẽ case, I would add to that list healthy social relations. My approach is consistent with the findings of Wolsko et al. (2006:345), who found that for the Yup’ik, notions of wellness “…emphasized the importance of living a traditional lifestyle, seeking creative solutions to manage drastic cultural change, and fostering connection within the communities and the native landscape.”

Among the A’uwẽ, many environmental dimensions may affect one’s well-being. A central theme of my approach to well-being is the cerrado landscape surrounding residential communities, which A’uwẽ people depend on for their subsistence, social and ceremonial activities, and ethnic identity. I do not engage with the prolific literature addressing relational schemata regarding “nature” and “culture” in Amazonia because such models were not borne out in my research as salient themes among the A’uwẽ (Descola 2013; Ingold 2006; Viveiros de Castro 1996). Although Barletti’s (2016) call to address nature/culture schemata as integral to Indigenous well-being may be pertinent and insightful for the Ashaninka and many other lowland South American groups, I did not perceive that such ontological relationships were of particular interest to the A’uwẽ. It has been noted that most Indigenous languages have no abstract terms that would support categories that separate nature from culture (e.g., nature, ecology, religion, animals, and plants) (Rival 2012). In the Xavante language spoken by the A’uwẽ, there are words for beautiful landscape (*rowedi*), spiritual ceremony (*wai’a*), animal (*abazé*), and plant (*ĩ’re*). This is not to say that the A’uwẽ are necessarily an exception to the tendency for Indigenous peoples to not categorically separate human society from the non-human landscape, but rather to suggest that they may not fit neatly into such relational schemata as they have been characterized previously. Currently, they have sophisticated concepts of the non-human environment as an anthropogenic place (shaped through time by gardening, burning, and occupation) and cultural resource (providing foods, materials, and useful spaces) that deserves conservation to ensure its resources remain abundant for future generations (Graham 2000; Welch 2014).

Considering social well-being as a process also suggests the importance of autonomy and sovereignty for making informed decisions in contexts of change, such as the A’uwẽ presently find themselves. The processes of change affecting the A’uwẽ are external, internal, and interconnected. They are rooted in deep to shallow timeframes, operate from the most global to local scales, and are recognized explicitly by all adult A’uwẽ. External changes that have occurred within the lifetimes of the women and men I met during my research range from the appearance and eventual insertion into Brazilian national society; sedentarization and monetarization of communities, and inclusion in regional and global networks of scholars, service providers, artists, and friends. This incomplete list illustrates how change is ubiquitous and its relationship to well-being is ambiguous. For example, many elders lament the loss of their former mobility as trekkers but celebrate their ability to have many healthy children and grandchildren due to local access to basic healthcare services and a sedentary lifestyle.

Contemporary lifestyle changes (formal schooling, digital technologies, bilingualism, monetary income, biomedicine, etc.) may be read by some as improvements mitigating against unwellness. At the same time, maintenance of social and environmental traditionalism may be interpreted as a source of ethnobiological resilience and strength, and therefore a source of wellness. From an anthropological point of view, just as suffering does not contradict well-being, neither does environmental change, for it is often precisely when conservation is needed that communities rally to preserve it (Lu 2005). Furthermore, efforts to restore environmental autonomy and sovereignty can contribute positively to well-being by improving the social conditions of life.

## Final comments

Antônio demonstrated the irreducibility and ambiguous nature of well-being when individual health and the social experience of wellness might be at odds with one another (Mathews and Izquierdo 2009). He also illustrated the intimate relationship between social dimensions of well-being and an environment undergoing transformation (Dasgupta 2001). The age group systems with oppositional and hierarchical as well as fraternal and collective aspects, described above, illustrate how social well-being is co-constructed by multiple segments of society that tend to prioritize traditionalism as expressed through certain conventional social roles and arrangements upheld as correct, beautiful, and respectful ways of living. Thus, from A’uwẽ perspectives, social relations of hierarchy and difference are integral to equality and collectivity, and all contribute through individual agency to social dimensions of wellness among community members. This local configuration of Indigenous perspectives regarding good living illustrates the processual aspect of collective social well-being, as individuals engage one another and through their interactions construct and reinforce shared notions of wellness.

My reading of A’uwẽ understandings of well-being direct me towards an analytical framework of social wellness as a heterogeneous process constructed by communities through shared understandings of forms of interpersonal relations potentially involving both pleasure and strife that are considered good and appropriate, being understood to accrue benefits for individuals, communities, and environments. This approach has the potential to rebalance debates about transcultural well-being indicators by redirecting attention to the emic societal contexts within and through which experiences of wellness and unwellness occur. This need has been well established for Aboriginal and Torres Strait Islander peoples and similar principles likely apply to Indigenous and other traditional peoples elsewhere (Butler et al. 2019). From a policy standpoint, this framework requires that well-being be considered holistically within socioculturally distinct settings and encourages recognition of peoples’ autonomy to understand health and well-being on their own terms and in relation to culturally relevant dimensions, including social life and local landscapes. It reinforces long established but rarely adequately attended policy viewpoints that health involves diverse dimensions of wellbeing, including social wellness (WHO 1948). It also has the potential to support community initiatives to increase well-being by strengthening its local cultural determinants, such as the social role of elders in an Indigenous community in Australia (Busija et al. 2020).

Underlying the ethnographic stories told in this article is the reality that not all youths share with their elders identical ideas about what behaviors are socially desirable and thereby contribute to living a good life. Younger women no longer abide being struck on their legs with wooden rods by their male mentors to test their strength. Younger contemporary fathers-in-law often invite their sons-in-law to disregard long established A’uwẽ conventions of expressing respect through avoidance behavior. Youths tend to prefer to purchase store-bought foods rather than grow, collect, hunt, and fish local foods that are preferred by many elders as important to a healthful diet and most valued as gifts of reciprocity between relatives and neighbors. Nevertheless, certain cultural events serve to anchor almost everyone’s perspectives regarding traditionalism and its linkages to well-being.

For example, the excitement of weddings motivates even those youths who are most repelled by the idea of hunting to enthusiastically participate in large group hunting expeditions undertaken to acquire ample quantities of game meat for the groom to deliver as a present to the doorstep of his parents-in-law’s house. They thereby learn hunting techniques and gain the capacity to provide sustenance for their families. Even young agnostics regarding A’uwẽ spirituality participate in each and every spiritual ritual, a challenging task, in order to lend their peers support and solidarity. With time, and their advancement through the spiritual ranks, they tend to become believers and gain capacities to heal their family members of disease and injury. Rites of passage into novitiate and mature adulthood are festive occasions in which the entire population participates in one way or another. They are occasions for previously unruly children to demonstrate respect for elders and commitment to their secular age set and age set moiety. These youths assume such responsibilities with solemnity and dedication, striving to demonstrate to the community their intentions to be good daughters and sons, mentors, children-in-law, spouses, and parents. In each of these examples, the appeal of participating in certain community social events brought youths into closer alignment with their elders in their perspectives of how to live well. Although what A’uwẽ understand to be traditional may change through time, certain social expressions of traditionalism unify otherwise disparate points of view and bring people together in their understandings of well-being and health.

In this article, I have sought to provide an ethnographic justification for a broader understanding of social aspects of well-being than is usually evident in mainstream multidisciplinary well-being literature. My argument only addresses a slice of the diverse considerations involved in a comprehensive treatment of well-being, which also include physical, mental, and spiritual dimensions, among others. Besides physical health, social configurations and relations were the dimensions that my A’uwẽ family, friends, consultants, and interlocutors emphasized the most in their conversations with me, making them ethnographically salient dimensions in my research. They also stood out as the aspects of well-being that most reflected A’uwẽ emphasis on local community, which is central to how they understand identity. Academic and policy attention to this perspective of well-being will contribute to an alignment with diverse local sociocultural realities, opening of space for two-way intercultural dialogue, and thereby also to the decolonialization of science and healthcare. A broad understanding of social well-being may also have concrete health impacts in diverse transcultural contexts, beyond Indigenous and culturally distinct peoples illustrated by the A’uwẽ case. For many localized and virtual communities impacted by adverse health events, such as Covid-19, forced migration, natural disaster, and economic crisis, restoring well-being requires more complex and holistic understandings of wellness to address non-physical sequelae, including restoring collective social well-being.

## Media teaser

Indigenous A’uwẽ (Xavante) notions of social well-being involve certain modes of suffering and inequality that are viewed as desirable for individuals and communities.
